# Device-Related Complications and Inappropriate Therapies Among Subcutaneous vs. Transvenous Implantable Defibrillator Recipients: Insight Monaldi Rhythm Registry

**DOI:** 10.3389/fcvm.2022.879918

**Published:** 2022-05-16

**Authors:** Vincenzo Russo, Anna Rago, Vincenzo Ruggiero, Francesca Cavaliere, Valter Bianchi, Ernesto Ammendola, Andrea Antonio Papa, Vincenzo Tavoletta, Stefano De Vivo, Paolo Golino, Antonio D'Onofrio, Gerardo Nigro

**Affiliations:** ^1^Cardiology Unit, Department of Medical Translational Sciences, University of Campania “Luigi Vanvitelli”, Monaldi Hospital, Naples, Italy; ^2^Cardiology Unit, Department of Cardiology, Monaldi – Hospital, Naples, Italy

**Keywords:** subcutaneous ICD (S-ICD), transvenous ICD, complications, infections, inappropriate shock therapy, mortality

## Abstract

**Introduction:**

In the context of randomized clinical trials, subcutaneous implantable cardiac defibrillators (S-ICDs) are non-inferior to transvenous ICDs (T-ICDs) concerning device-related complications or inappropriate shocks in patients with an indication for defibrillator therapy and not in need of pacing. We aimed at describing the clinical features of patients who underwent S-ICD implantation in our clinical practice, as well as the ICD-related complications and the inappropriate therapies among S-ICD vs. T-ICD recipients during a long-term follow-up.

**Materials and Methods:**

All patients undergoing ICD, both S-ICD and TV-ICD, at Monaldi Hospital from January 1, 2015 to January 1, 2019 and followed up at our institution were included in the present analysis. The clinical variables associated with S-ICD implantation were evaluated by logistic regression analyses. We collected the ICD inappropriate therapies, ICD-related complications (including both pulse generator and lead-related complications), ICD-related infections, appropriate ICD therapies, and overall mortality. Kaplan-Meier (KM) analyses were performed to assess the risk of clinical outcome events between the two subgroups. A time-dependent Cox regression analysis was performed to adjust the results.

**Results:**

Total 607 consecutive patients (mean age 53.8 ± 16.8, male 77.8%) with both TV-ICD (*n*: 290, 47.8%) and S-ICD (*n*: 317, 52.2%), implanted and followed at our center for a mean follow-up of 1614 ± 1018 days, were included in the study. At multivariate logistic regression analysis, an independent association between S-ICD implantation and ionic channel disease [*OR*: 6.01 (2.26–15.87); *p* < *0.0001*] and ischemic cardiomyopathy [*OR*: 0.20 (0.12–0.35); *p* < *0.0001*] was shown. The KM analysis did not show a significantly different risk of the inappropriate ICD therapies (*log rank p* = *0.64*) between the two subgroups; conversely, a significant increase in the risk of ICD-related complications (*log rank p* = *0.02*) and infections (*log rank p* = *0.02*) in TV-ICD group was shown. The adjusted risk for ICD-related infections [*OR*: 0.07 (0.009–0.55), *p* = *0.01*] and complications [0.31 (0.12–0.81)*, p* = *0.01*] was significantly lower among patients with S-ICD.

**Conclusions:**

The choice to implant S-ICD was mainly driven by younger age and the presence of ionic channel disease; conversely ischemic cardiomyopathy reduces the probability to use this technology. No significant differences in inappropriate ICD therapies were shown among S-ICD vs. TV-ICD group; moreover, S-ICD is characterized by a lower rate of infectious and non-infectious complications leading to surgical revision or extraction.

## Introduction

The subcutaneous implantable cardioverter-defibrillator (S-ICD) is an established therapy for the prevention of sudden cardiac death (SCD) (1) and an alternative to a transvenous implantable cardioverter-defibrillator (T-ICD) system in selected patients (2). S-ICD may be particularly useful in patients with channelopathies (3) since several studies showed a high complication rate in those implanted with T-ICD (4–6). S-ICD is non-inferior to T-ICD concerning device-related complications or inappropriate shocks in patients with an indication for defibrillator therapy and not in need of pacing (7–12); however, these data are limited to short follow-up observational case-control studies (7–10) or the context of the randomized clinical trial (11, 12). Moreover, few data about the clinical drivers of S-ICD vs. T-ICD implantation in clinical practice have been still provided. We therefore aimed at describing the clinical features of patients who underwent S-ICD implantation, as well as the ICD-related complications and the inappropriate therapies among S-ICD vs. T-ICD recipients in the clinical practice of a high-volume implantation center.

## Materials and Methods

### Database

Data for this study were sourced from the Monaldi Hospital Rhythm Registry (NCT05072119), which includes all patients who underwent ICD implantation and followed up at our Institution through both outpatient visits, every 3–6 months, and remote device monitoring. During the follow-up, the occurrence and the causes of inappropriate and appropriate ICD therapies, ICD-related complications, and deaths were assessed and recorded in the electronic data management system. For the present analysis, we selected all consecutive patients who received *de novo* both subcutaneous (S-ICD Group) and transvenous (TV Group), from January 1, 2015 to January 1, 2019, according to the European guidelines and recommendations available at the time of implantation (13, 14). We excluded patients with pacing indications (*n*: 87), CRT (*n*: 232), upgrade of an existing device (*n*: 56), incomplete baseline (*n*: 36) or follow-up data (*n*: 48). The local institutional review boards approved the study (ID 553-19), and all patients provided written informed consent for data storage and analysis.

### ICD Programming

The programming of the parameters for the detection of VT/VF was done according to the guidelines recommendations at the time of implant. In particular, we routinely activate for primary prevention only one VF zone (30 intervals at 250 bpm) and for secondary prevention two windows of detection (VF: 30 intervals at 250 bpm; VT2: 30 intervals at 187 bpm or 10–20 bpm < VT rate) with shocks only in VF zone and up to three ATPs and eight shocks in VT2 zone. S-ICD devices were programmed with a conditional zone, between 200 and 250 bpm, and a shock zone > 250 bpm. The programmed sensing vector was primary (48.42%) or secondary (43.4%) for most patients and alternate in a small percentage of cases (8.18%). The bicycle ergometer test was not routinely performed in patients who implanted S-ICD at our Hospital.

### Outcomes

The primary study endpoints were: ICD inappropriate therapies, defined as anti-tachycardia pacing (ATP) and/or shocks for conditions other than ventricular tachycardia (VT) or ventricular fibrillation (VF); ICD-related complications, defined as all pulse generator (PG) or lead-related complications requiring surgical intervention; ICD-related infections, defined as all systemic infections requiring complete removal of the system including the leads extraction. The secondary endpoints were the clinical variables associated to S-ICD implantation, appropriate ICD therapies and all-cause mortality. Moreover, the type and distribution of ICD-related complications, defined as early when appearing during the first 30 days after device implantation or late, when occurred after the first-month post-implantation, were assessed.

### Statistical Analysis

Categorical data were expressed as number and percentage, whereas continuous variables were expressed as either median [interquartile range (*IQR*)]) or mean ± SD, based on their distribution as assessed both by the Kolmogorov–Smirnov and the Shapiro–Wilk tests. Between-group differences, for categorical variables, were assessed by the chi-square test, with the application of Yates correction where appropriate. Either parametric Student's *t*-test or nonparametric Mann–Whitney U test and Wilcoxon test were instead used to compare continuous variables, according to their distribution. Kaplan–Meier analysis was performed to assess the risk of both inappropriate ICD therapies and ICD-related complications between the two subgroups. Univariate and multivariate logistic regression was used to assess the clinical characteristics associated with S-ICD implantation. A time-dependent Cox univariate (unadjusted) and multivariate (adjusted) regression model was used to evaluate the association between S-ICD and clinical outcome events. The multivariate model was computed on all covariates with a *p*-value < 0.05. A 2-sided probability *p*-value < 0.05 was considered statistically significant. All analyses were performed using SPSS statistical software (version 24.0, SPSS, Chicago, Illinois) and STATA 14.0 software (StataCorp, College Station, Texas).

## Results

### General Characteristics of the Study Population

A total of 607 consecutive patients (mean age 53.8 ± 16.8, male 77.8%) with both TV-ICD (*n*: 290, 47.8%) and S-ICD (*n*: 317, 52.2%) followed at our center for a mean follow-up of 1,614 ± 1,018 days were included in the study. The indication for ICD implantation was primary prevention in 542 patients (89%) and secondary prevention in 65 patients (11%). S-ICD group showed more likely younger age (49 ± 17 vs. 60 ± 14 years; *p*< * 0.0001*), higher left ventricular ejection fraction (LVEF) (41 ± 17 vs. 35 ± 12 %; *p* < * 0.0001*), and lower prevalence of cardiovascular comorbidities. Ischemic cardiomyopathy (44.5 vs. 27%; *p* < * 0.0001*) and idiopathic dilated cardiomyopathy (32.4 vs. 17.3 %; *p* < * 0.0001*) were more frequent in the TV-ICD group; conversely, ionic channel disorders (16.7 vs. 2.75 %; *p* < * 0.0001*) were more frequent in S-ICD group. The ionic channel disorders group included patients with long QT syndrome (*n*: 8) and Brugada syndrome (*n*: 53). All baseline clinical characteristics of the study population are summarized in Table 1.

**Table 1:** Baseline characteristics of the study population.

**Parameter**	**TV-ICD group** ***n: 290***	**S-ICD group** ***n: 317***	***p***
Male gender, *n* (%)	228 (79)	239 (75)	0.24
Age (years), mean ± SD	60 ± 14	49 ± 17	<0.0001
LVEF (%), mean ± SD	35 ± 12	41 ± 17	<0.0001
Idiopathic dilated cardiomyopathy, *n* (%)	94 (32.4)	55 (17.3)	<0.0001
Ischemic cardiomyopathy, *n* (%)	129 (44.5)	86 (27)	<0.0001
Hypertrophic cardiomyopathy, *n* (%)	29 (10)	48 (15)	0.06
ARVD, *n* (%)	3 (1)	10 (3)	0.08
Ionic channel disorders, *n* (%)	8 (2.75)	53 (16.7)	<0.0001
Primary prevention*, n* (%)	239 (82.4)	303 (95)	<0.0001
Secondary prevention*, n* (%)	51 (17.5)	14 (4.4)	<0.0001
NYHA I, *n* %	15 (5)	55 (17.3)	<0.0001
NYHA II, *n* %	151 (52)	123 (38.8)	0.001
NYHA III, *n* %	105 (36)	74 (23.3)	0.0006
NYHA IV, *n* %	19 (7)	2 (0.6)	<0.0001
Hypertension, *n* (%)	190 (65.5)	93 (29)	<0.0001
Diabetes, *n* (%)	87 (30)	38 (12)	<0.0001
COPD, *n* (%)	41 (14)	45 (14)	1
CAD, *n* (%)	121 (41.7)	79 (25)	<0.0001
AF history, *n* (%)	72 (24.8)	44 (13.9)	0.0006
CKD, *n* (%)	46 (16)	34 (11)	0.07
Previous valve replacement, *n* (%)	17 (5.8)	15 (4.7)	0.54
Previous CABG, *n* (%)	22 (7.5)	22 (6.9)	0.77

*LVEF, left ventricular ejection fraction; ARVD, arrhythmogenic right ventricular dysplasia; NYHA, New York Heart Association; COPD, chronic obstructive pulmonary disease, CAD, coronary artery disease; CKD, Chronic Kidney Disease; AF, Atrial fibrillation; CABG, Coronary Artery Bypass Graft*.

### Clinical Variables Associated With S-ICD Implantation

We assessed potential clinical variables associated with S-ICD implantation among our study population. At multivariate logistic regression analysis, an independent association between S-ICD implantation and ionic channel disease [*OR*: 6.01 (2.26–15.87); *p* < *0.0001*], ischemic cardiomyopathy [*OR:* 0.20 (0.12–0.35); *p* < *0.0001*] was shown. All data are shown in Table 2.

**Table 2:** Association between S-ICD implantation and clinical covariates: univariate and multivariate analysis.

	**Univariate Analysis** **OR [95% CI]**	***p***	**Multivariate Analysis** **OR [95% CI]**	***p***
Male gender	0.91 [0.62–1.35]	0.65	-	-
Age	0.96 [0.96–0.98]	<0.0001	0.99 [0.98–1.01]	0.11
LVEF	1.03 [1.02–1.04]	<0.0001	0.98 [0.97–1.01]	0.09
Idiopathic dilated cardiomyopathy	0.44 [0.31–0.64]	<0.0001	0.80 [0.42–1.56]	0.5
Ischemic cardiomyopathy	0.46 [0.33–0.65]	<0.0001	0.20 [0.12–0.35]	<0.0001
Hypertrophic cardiomyopathy	1.62 [0.99–2.64]	0.06	*-*	-
ARVD	3.12 [0.85–11.44]	0.09	*-*	-
Ionic channel disorders	7.07 [3.30–15.16]	<0.0001	6.01 [2.26–15.87]	<0.0001
Hypertension	0.23 [0.16–0.32]	<0.0001	0.62 [0.27– 1.13]	0.25
Diabetes	0.33 [0.22–0.51]	<0.0001	0.55 [0.34–1.07]	0.08
COPD	1.03 [0.65–1.63]	0.8	*-*	-
CAD	0.48 [0.34–0.67]	<0.0001	0.60 [0.34–1.12]	0.12
CKD	0.66 [0.41–1.05]	0.08	*-*	-
AF history	0.49 [0.32–0.74]	0.007	0.67 [0.41–1.09]	0.11
Previous valve replacement	0.81 [0.40–1.65]	0.55	*-*	-
Previous CABG	0.94 [0.51–1.73]	0.83	*-*	-

*LVEF, left ventricular ejection fraction; ARVD, arrhythmogenic right ventricular dysplasia; COPD, chronic obstructive pulmonary disease; CAD, coronary artery disease; CKD, Chronic Kidney Disease; AF, Atrial fibrillation; CABG, Coronary Artery Bypass Graft*.

### Clinical Outcomes Between the Groups

#### Inappropriate ICD Therapies

**Figure 1:**
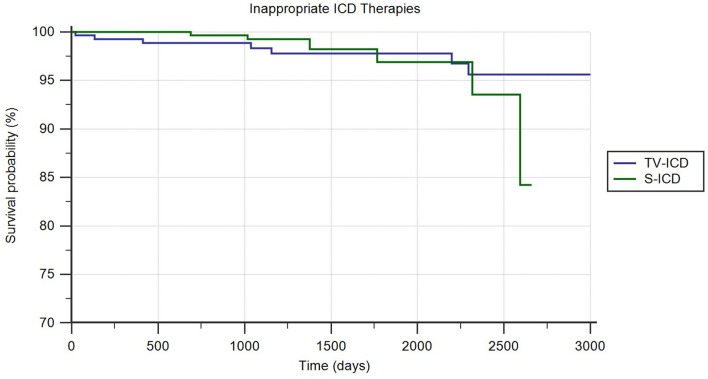
Kaplan-Meier curve comparing survival without ICD-related infections among S-ICD vs. TV-ICD groups.

Among our study population, ICD inappropriate therapies were experienced by 14 patients (2.31%). Out of these, seven patients (2.41%) in the TV-ICD group and seven patients (2.2%) in S-ICD group (*p* = *0.56*). The annual incident rate of ICD inappropriate therapies over the follow-up was 0.6%. The Kaplan–Meier analysis did not show a significantly different risk of inappropriate ICD therapies between the two subgroups (*log-rank p* = *0.64*) (Figure 1). At Cox univariate analysis no baseline patients' characteristic, including the S-ICD (*OR*: 1.30; 95% *CI*: 0.43–3.96; *p* = *0.64*), was associated with inappropriate ICD therapies (Supplementary Table 1).

#### ICD-Related Complications

**Figure 2:**
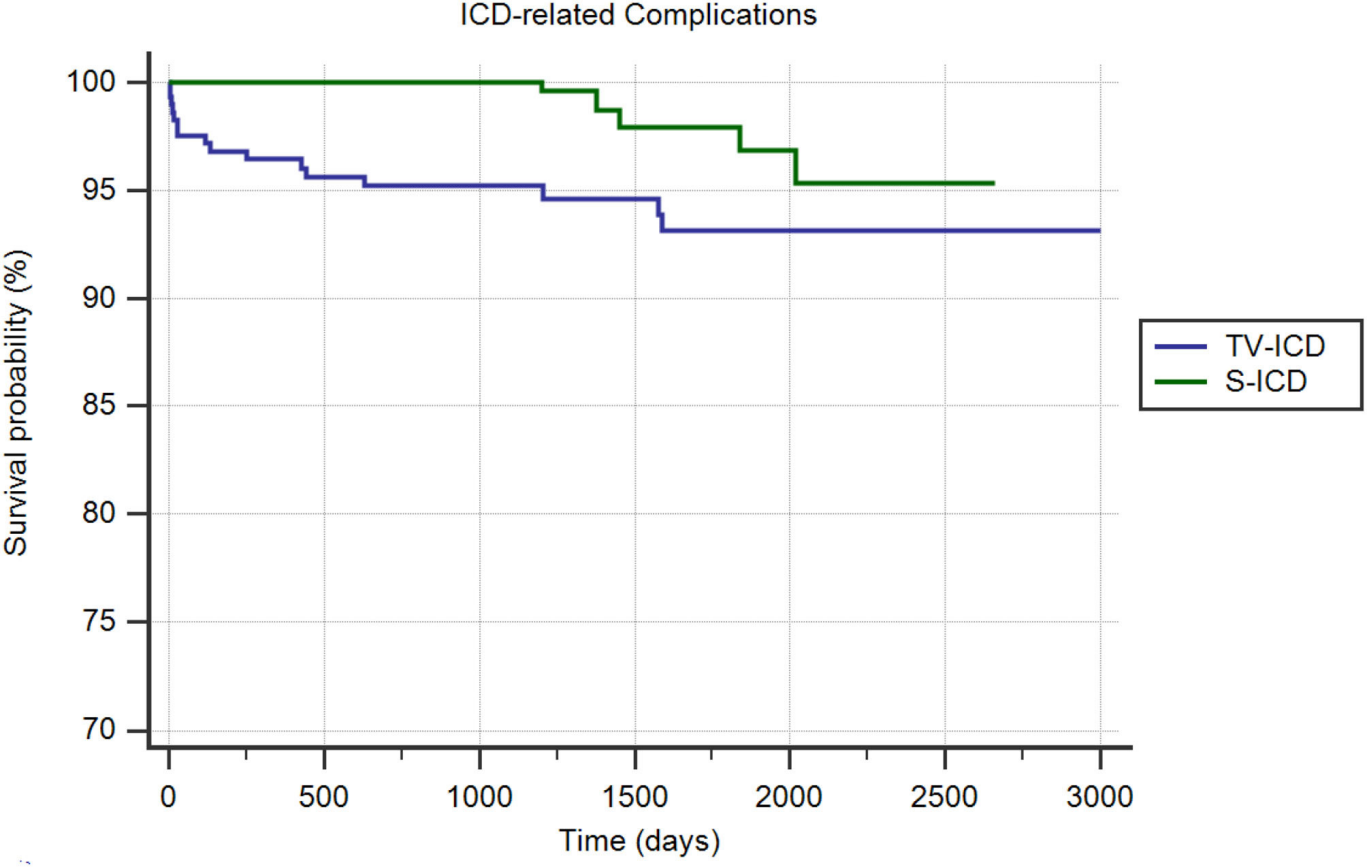
Kaplan-Meier curve comparing survival without ICD related complications among S-ICD vs. TV-ICD groups.

ICD related complications in need of surgical revision occurred in 24 patients (3.9%); 18 (6.2%) in TV-ICD group and 6 (1.9%) in S-ICD group (*p* = *0.006*); mainly due to increased lead-related complications in TV-ICD vs. S-ICD group (5.9 vs. 0.3%; *p* = *0.001)*. In contrast, no significant differences were shown in PG-related complications between the two subgroups (0.34 vs. 1.72%; *p* = *0.09)*. The Kaplan–Meier analysis showed a significantly increased risk of ICD-related complications among the TV-ICD group (*log-rank p* = *0.02*) (Figure 2). At Cox multivariate analysis, S-ICD was the only variable significantly associated with a reduction of ICD-related complications (*OR*: 0.31; 95% *CI* 0.12–0.83; *p* < *0.01)* (Supplementary Table 2).

#### ICD-Related Infections

**Figure 3:**
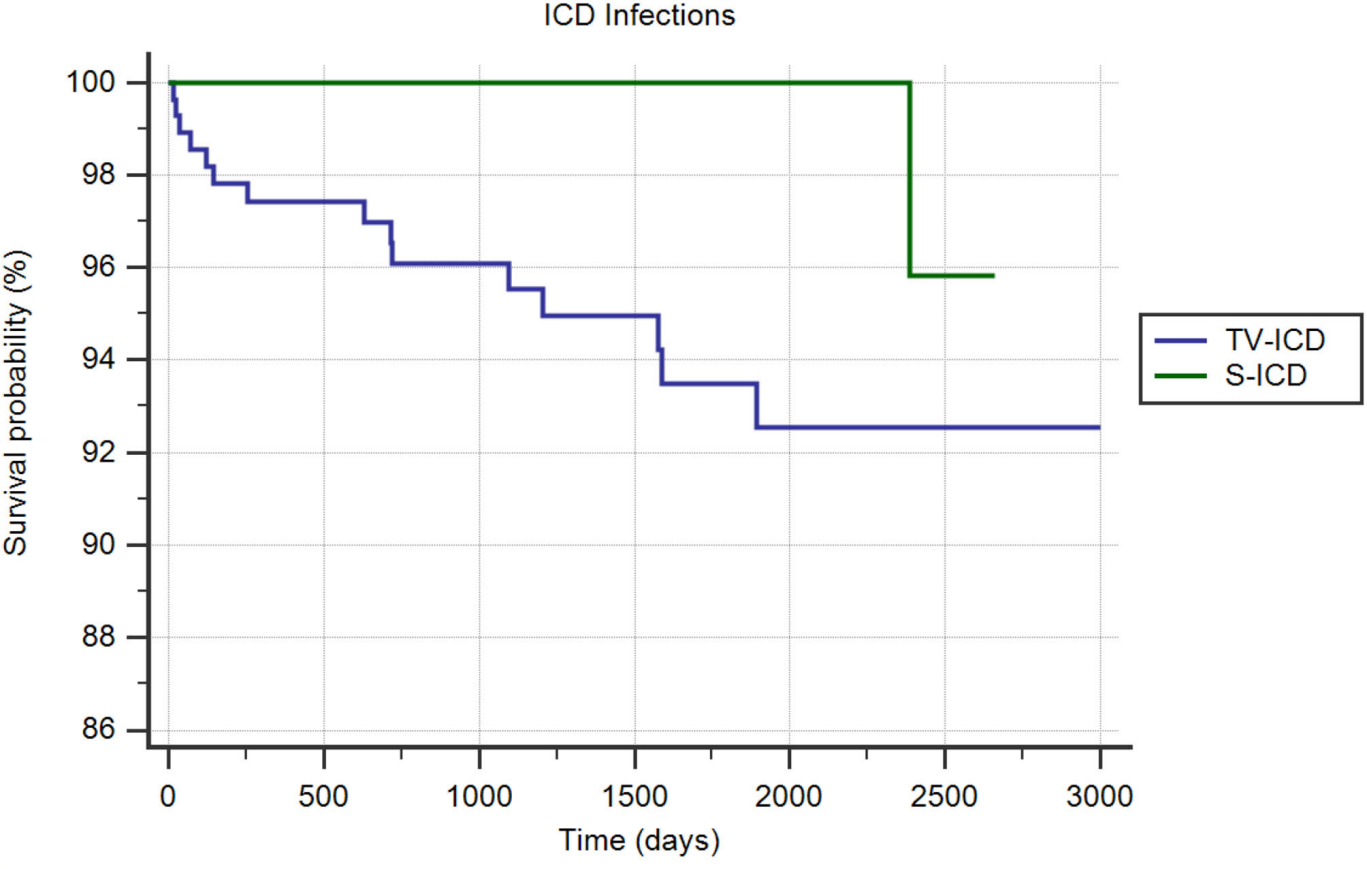
Kaplan-Meier curve comparing survival without ICD infections among S-ICD vs. TV-ICD groups.

ICD-related infections in need of leads extraction occurred in 11 patients (1.8%); 10 (3.4%) in TV-ICD group and 1 (0.3%) in S-ICD group (*p* = *0.004*). The annual incident rate of ICD-related infections over the follow-up was 0.4%. The Kaplan-Meier analysis showed a significantly increased risk of ICD-related infections among the TV-ICD group (*log-rank p* = *0.02*) (Figure 3). At Cox multivariate analysis, S-ICD was the only variable significantly associated with a reduction of ICD-related infections (*OR*: 0.07; 95% *CI* 0.009–0.55; *p* < *0.01*); in contrast previous valve replacement (*OR*: 7.22; 95% *CI* 2.34–22.22; *p* = *0.0006*) was associated with an increased risk of ICD-related infections (Supplementary Table 3).

In the Table 3 were summarized all primary outcomes events at follow-up among the two subgroups.

**Table 3:** Primary outcome events at follow-up.

**Parameter**	**TV-ICD group** ***n = 290***	**S-ICD group** ***n = 317***	***p***
Inappropriate ICD therapies, *n* (%)	7 (2.4)	7 (2.2)	0.65
Inappropriate shock, *n* (%)	4 (1.37)	7 (2.2)	0.44
Inappropriate ATP, *n* (%)	3 (1)	0 (0)	0.07
Causes of inappropriate therapies
T wave oversensing, *n* (%)	0 (0)	4 (1.3)	0.05
Myopotential oversensing, *n* (%)	0 (0)	2 (0.6)	0.19
Atrial fibrillation, *n* (%)	5 (1.7)	0 (0)	0.02
Atrial tachycardia, *n* (%)	2 (1.37)	1 (0.3)	0.14
ICD related complications, *n* (%)	18 (6.2)	6 (1.9)	0.007
PG related complications, *n* (%)	1 (0.34)	5 (1.72)	0.09
PG Malfunction, *n* (%)	1 (0.34)	5 (1.72)	0.09
Lead related complications, *n* (%)	17 (5.9)	1 (0.3)	<0.0001
Lead failure, *n* (%)	5 (2)	0 (0)	0.01
Lead dislodgement, *n* (%)	2 (0.7)	0 (0)	0.14
Lead Fracture, *n* (%)	10 (3.4)	1 (0.3)	0.004
ICD infectious complications	10 (3.4)	1 (0.3)	0.004
Timing of overall complications
Early complications	8 (2.75)	0 (0)	0.003
Late complications	20 (6.9)	7 (2.2)	0.005

#### Appropriate ICD-Therapies

Among our study population, ICD appropriate therapies were experienced by 56 patients (9.23%). Out of these, 46 patients (15.86%) in the TV-ICD group and 10 patients (3.15%) in S-ICD group (*p* = *0.0001*). Table 4 shows the number and the underlying disease of patients with at least one appropriate ICD therapy among our study population. The annual incident rate of ICD appropriate therapies over the follow-up was 2.3%. The Kaplan–Meier analysis showed a significantly increased risk of appropriate ICD therapies among the TV-ICD group (*log-rank p* = *0.04*). At Cox multivariate analysis, no clinical variables were independently associated with an increased risk of ICD appropriate therapy (Supplementary Table 4).

**Table 4:** Number of patients with at least one appropriate ICD therapy across different patients subgroups.

	**TV-ICD group**		**S-ICD group**
	**Shock**	**ATP**	**Shock**
Idiopathic dilated cardiomyopathy, *n*	7	11	3
Ischemic cardiomyopathy, *n*	9	11	2
Hypertrophic cardiomyopathy, *n*	3	2	1
Brugada syndrome, *n*	1	0	1
LQTS, *n*	0	0	2
ARVD, *n*	1	0	1

*LQTS, Long QT syndrome; ARVD, arrhythmogenic right ventricular dysplasia*.

#### All-Cause Mortality

**Figure 4:**
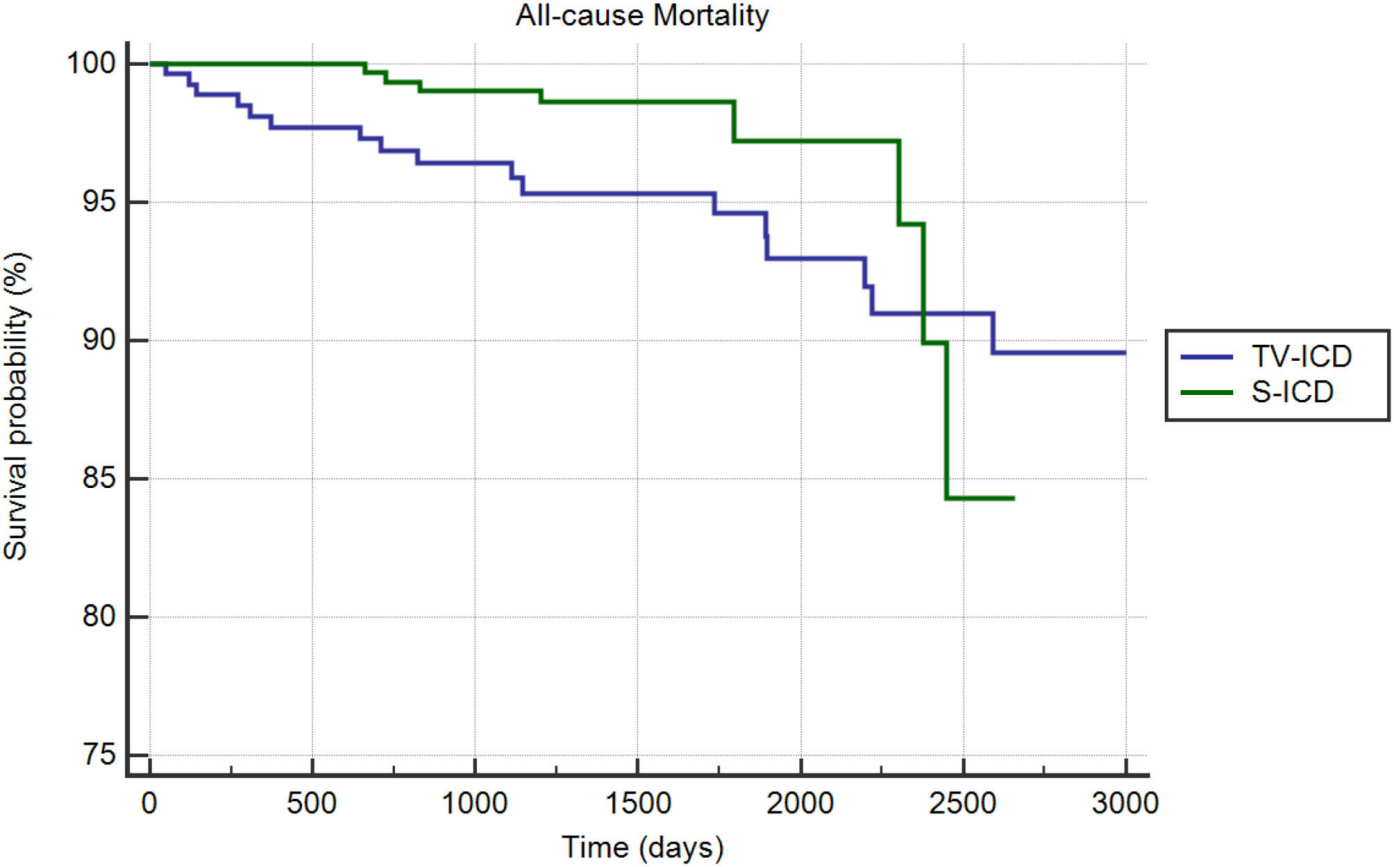
Kaplan-Meier curve comparing survival without all-cause mortality among S-ICD vs. TV-ICD groups.

During the follow-up period, 28 people (4.61 percent) died: 8 patients (2.52%) in the S-ICD group and 20 (6.9%) in the TV-ICD group (*p* = *0.01*). The annual incident rate of mortality over the follow-up was 1.15%. The Kaplan–Meier analysis did not show a significantly different risk of death between the two groups (*log-rank p* = *0.52*) (Figure 4). At Cox multivariate analysis, no clinical variables were independently associated with all-cause mortality (Supplementary Table 5). Table 5 shows the unadjusted and adjusted odds ratio for the association between the clinical outcomes of interest and S-ICD.

**Table 5:** Unadjusted and adjusted odds ratio for S-ICD and the clinical outcomes of interest.

	**Unadjusted OR [95% CI],** ***p***	**Adjusted OR [95% CI],** ***p***
Inappropriate ICD therapies	1.30 [0.43–3.96], 0.64	-
ICD related infections	0.05 [0.007–0.44], 0.006	0.07 [0.009–0.55], 0.01*
ICD related Complications	0.32 [0.12–0.83], 0.01	0.31 [0.12–0.81], 0.01**
Appropriate ICD therapies	0.46 [0.21–0.98], 0.04	0.54 [0.25–1.18], 0.12***
Overall-Mortality	0.89 [0.38–2.09], 0.79	-

* *Adjusted for ischemic cardiomyopathy, chronic kidney disease, and previous valve replacement*.

** *Adjusted for age and sex*.

*** *Adjusted for left ventricular ejection fraction, arrhythmogenic right ventricular dysplasia, Ionic channel disorders, diabetes, chronic obstructive pulmonary disease*.

## Discussion

The main results of our study are the following: younger age and ionic channel diseases are clinical variables independently associated with S-ICD implantation for sudden cardiac death prevention; conversely, ischemic cardiomyopathy reduced the probability to receive S-ICD among our study population. S-ICD patients showed a lower rate of both ICD-related complications and infections and no significant differences in inappropriate ICD therapies compared to TV-ICD patients during the follow-up. Finally, no differences inappropriate ICD therapies and overall mortality have been shown between the two groups. The lower age of the S-ICD group and the higher prevalence of ionic channel disease as clinical drivers of S-ICD implantation among our study population confirm the tendency to consider S-ICD the preferred choice for patients with an active lifestyle and long-life expectance. This is particularly true for inherited genetic arrhythmogenic syndromes (Brugada Syndrome and Long QT syndrome) where clinical arrhythmias are polymorphic VT or VF (not treatable with ATP) and the risk of bradycardia and monomorphic VT is very low (15, 16). The reduced probability for patients with ischemic cardiomyopathy to receive an S-ICD might be due to fair of sustained VT in need of anti-tachycardia pacing (ATP) or incident bradyarrhythmias in need of pacing (17). However, it should be noted that only 15–20% of patients experienced a high rate of monomorphic VT during the first year after the implant with a subsequent risk is 1.8%/year; moreover, the proportions of both monomorphic VT and successful ATP was comparable between patients with ischemic and non-ischemic cardiomyopathy (18). Finally, no studies have still addressed whether the efficacy of ATP translates into hard outcomes such as mortality benefits, prevention of inappropriate shocks, and risks of pro-arrhythmias (19).

Based on this evidence, the choice to implant an ATP-capable ICD should not exclusively be based on the ischemic or non-ischemic cardiomyopathy, but it should have applied a patient's centered tailoring approach which takes into account the potential mechanisms of ventricular arrhythmias and other patient factors such as susceptibility to systemic infections. Our study population included a large cohort of patients with HCM who were more likely treated with S-ICD; this preferred choice may be justified by the low rate of ATP therapies experienced by patients with HCM, with no difference in the rate of shock therapy compared to those with TV-ICD (20). However, the choice to implant an S-ICD should take into account the clinical features of patients with HCM since older age and symptomatic patients seem to be more likely to benefit from T-ICD pacing for the high incidence of symptomatic bradycardia and conduction disturbances in need of pacing, together with monomorphic ventricular tachycardia, as the predominant rhythm triggering successful ATP therapy (21). Similarly regarding ARVD, TV-ICD should be preferred in older patients with an advanced form of the disease, who more often experienced re-entrant VT that could be interrupted by ATP; in contrast, S-ICD is more indicated among younger patients who more likely experienced VF and are particularly prone to lead-related complications requiring device explantation (22). Recently, the prospective randomized comparison of subcutaneous and transvenous implantable cardioverter-defibrillator therapy (PRAETORIAN) trial (12) showed that, among 849 patients with an indication for ICD therapy but not for pacing therapy, the S-ICD was non-inferior to the T-ICD concerning the cumulative incidence of device-related complications or inappropriate shocks. However, there was a higher cumulative incidence of device-related complications in the T-ICD group (9.8 vs. 5.9%) and a higher cumulative incidence of inappropriate shocks in the S-ICD group (9.7 vs. 7.3%) at a median duration of follow-up was 49.1 months. Moreover, S-ICD was associated with a lower risk of lead-related complications, which was counter balanced by an increased risk of pocket hematoma.

A recent metanalysis of 13 randomized clinical trials including 9,073 patients (10) showed that the overall risk of clinically relevant complications and inappropriate shocks was not different between patients treated with S-ICD and TV-ICD. On the contrary, the risk of lead-related complications and major procedural complications was higher in the TV-ICD arm. No significant differences were found in the incidence of appropriate shocks and mortality was comparable between the two devices.

Among our study population, the cumulative incidence of inappropriate therapies was lower than previously reported, mainly due to our strategy to optimize the TV-ICD programming at each follow-up visit or based on remote monitoring reporting. In particular, an approach based on the programming of a VF-only zone (23), a cut-off rate greater than 220–240 bpm (24), longer detection intervals (25), activation of lead noise reduction algorithms (26), and enhanced supraventricular tachycardia discriminators (27) was used in our clinical practice. Moreover, the generation of S-ICD systems implanted at our Institution (EMBLEM A209 and EMBLEM-MRI A219) can apply an additional high-pass filter to the sensing methodology, called SmartPass (SP), designed to reduce inappropriate therapies (28). As previously shown in PRETORIAN trial, we did not observe a significant difference in the cumulative incidence of inappropriate therapies between the S-ICD and TV-ICD groups. The main cause of inappropriate therapies was oversensing in S-ICD group and misdetection of supraventricular arrhythmias in the TV-ICD group.

Regarding the complications, we observed a significant reduction of overall ICD-related complications in the S-ICD group, mainly driven by less frequent lead-related complications; in contrast, the device-related complications were higher in the S-ICD group due to some advisory released by Boston Scientifics for generators.^1^

Among our population, we reported a low annual rate of ICD infections, confirming the reduced number of infections in high implantation volume centers (29, 30); as we expected, the TV-ICD group showed higher incidence compared to the S-ICD group. This evidence is of pivotal importance since systemic infections represent an important predictor of death for all causes, regardless of the result of the extraction procedure (31).

### Study Limitations

The present is a single-center observational study mainly including ICD recipients, both TV-ICD and S-ICD, not in need of pacing and CRT. As we expected, the baseline clinical characteristics of the two subgroups were different and a regression analysis was performed to identify which variables have impact on the outcomes of interest; however, due to the observational nature of the study, we cannot exclude residual confounding of unmeasured variables. The results of the present study might be influenced by the high experience in ICD implantation and management of our center. The follow-up is relatively short, about 48 months, however, it is the longest among observational studies. No data about pharmacological therapies have been collected at the time of outcomes events.

## Conclusions

In our clinical practice, the choice to implant S-ICD has been mainly driven by younger age and the presence of ionic channel disease; conversely, ischemic cardiomyopathy reduces the probability to use this technology. There were no significant differences in inappropriate ICD therapies between S-ICD and TV-ICD group; moreover, S-ICD has a lower rate of infectious and non-infectious complications leading to surgical revision or extraction.

## Data Availability Statement

The raw data supporting the conclusions of this article will be made available by the authors, without undue reservation.

## Ethics Statement

The studies involving human participants were reviewed and approved by University of Campania - Monaldi Hospital. The patients/participants provided their written informed consent to participate in this study.

## Author Contributions

VRus designed the study. AR, EA, AP, VT, VB, and SD collected the data. VRug, VRus, and FC performed statistical analysis. VRus wrote the manuscript. AD'O, PG, and GN performed critical revision of article. All authors read and revised the manuscript.

## Funding

The principal investigator, as senior researcher at Department of Medical Translational Sciences of University of Campania, received an unrestricted research grant from Boston Scientific. The funder was not involved in the study design, collection, analysis, interpretation of data, the writing of this article or the decision to submit it for publication.

## Conflict of Interest

The authors declare that the research was conducted in the absence of any commercial or financial relationships that could be construed as a potential conflict of interest.

## Publisher's Note

All claims expressed in this article are solely those of the authors and do not necessarily represent those of their affiliated organizations, or those of the publisher, the editors and the reviewers. Any product that may be evaluated in this article, or claim that may be made by its manufacturer, is not guaranteed or endorsed by the publisher.

## Footnotes

^1^Available online at: https://www.bostonscientific.com/content/dam/bostonscientific/quality/dlt/reg-code-228/2020Dec_BSC_EmblemPBD_PhysLtr_US_Final.pdf.
