# Supplementary material for: Device-Related Complications and Inappropriate Therapies Among Subcutaneous vs. Transvenous Implantable Defibrillator Recipients: Insight Monaldi Rhythm Registry
Source: Front Cardiovasc Med. 2022 May 16;9:879918. doi: 10.3389/fcvm.2022.879918 (PMC9150501; doi:10.3389/fcvm.2022.879918)
Supplement: Supplementary file 1 [file Data_Sheet_1.docx]

**Supplemental Table 1:** Association between inappropriate ICD therapies and baseline patients‘ characteristics

|  | **Univariate Analysis**  **OR [95% CI]** | ***P*** | **Multivariate Analysis**  **OR [95% CI]** | ***P*** |
| --- | --- | --- | --- | --- |
| Male gender | 0.68 [0.21- 2.18] | *0.52* | *-* | *-* |
| Age (years) | 0.99[0.96-1.03] | *0.89* | *-* | *-* |
| LVEF (%) | 1.03[0.99-1.07] | *0.09* | *-* | *-* |
| Idiopathic dilated cardiomyopathy | 1.06 [0.33- 3.38] | *0.93* | *-* | *-* |
| Ischemic cardiomyopathy | 0.83 [0.26- 2.65] | *0.75* | *-* | *-* |
| Hypertrophic cardiomyopathy | 0.88 [0.19- 3.98] | *0.87* | *-* | *-* |
| ARVD | 0.98 [0.22- 2.78] | *0.96* | *-* | *-* |
| Ionic channel disorders | 0.59 [0.07- 4.54] | *0.61* | *-* | *-* |
| Atrial fibrillation | 2.21 [0.73-6.66] | *0.16* | *-* | *-* |
| Hypertension | 0.54 [0.18- 1.66] | *0.28* | - | *-* |
| Diabetes | 0.53 [0.12- 2.40] | *0.42* | *-* | *-* |
| COPD | 0.94 [0.21- 4.22] | *0.94* | *-* | *-* |
| CAD | 0.88 [0.28- 2.81] | *0.83* | *-* | *-* |
| CKD | 1.81 [0.50- 6.51] | *0.36* | *-* | *-* |
| Previous valve replacement | 2.78 [0.36- 21.62] | *0.32* | *-* | *-* |
| Previous CABG | 0.89 [0.18- 2.91] | *0.98* | *-* | *-* |
| AF History | 2.21[0.73-6.66] | *0.16* | - | *-* |
| S-ICD | 1.30 [0.43-3.96] | *0.64* | - | *-* |

**Supplemental Table 2:** Association between ICD related complications and baseline patients ‘characteristics

|  | **Univariate Analysis**  **OR [95% CI]** | ***P*** | **Multivariate Analysis**  **OR [95% CI]** | ***P*** |
| --- | --- | --- | --- | --- |
| Male gender | 0.99 [0.36- 2.70] | *0.99* | *-* | *-* |
| Age (years) | 0.99 [0.97-1.02] | *0.71* | *-* | *-* |
| LVEF (%) | 0.99 [0.97-1.02] | *0.95* | *-* | *-* |
| Idiopathic dilated cardiomyopathy | 1.00 [0.39-2.52] | *0.99* | *-* | *-* |
| Ischemic cardiomyopathy | 1.46 [0.64-3.31] | *0.35* | *-* | *-* |
| Hypertrophic cardiomyopathy | 1.10 [0.37-3.29] | *0.85* | *-* | *-* |
| ARVD | 0.92 [0.28- 2.51] | *0.96* | *-* | *-* |
| Ionic channel disorders | 0.32 [0.06- 1.651] | *0.86* | *-* | *-* |
| AF History | 0.34 [0.07- 1.48] | *0.15* | *-* | *-* |
| Hypertension | 1.24 [0.55- 2.81] | *0.59* | - | *-* |
| Diabetes | 0.73 [0.24- 2.15] | *0.57* | *-* | *-* |
| COPD | 1.48 [0.55- 3.96] | *0.43* | *-* | *-* |
| CAD | 1.55 [0.68- 3.50] | *0.29* | *-* | *-* |
| CKD | 1.44 [0.49- 4.25] | *0.50* | *-* | *-* |
| Previous Valve replacement | 0.91 [0.25- 3.89] | *0.98* | *-* | *-* |
| Previous CABG | 0.55 [0.07- 4.09] | *0.56* | *-* | *-* |
| S-ICD | 0.32 [0.12- 0.83] | *0.01* | *-* | *-* |

**Supplemental Table 3:** Association between ICD related infections and baseline patients ‘characteristics

|  | **Univariate Analysis**  **OR [95% CI]** | ***P*** | **Multivariate Analysis**  **OR [95% CI]** | ***P*** |
| --- | --- | --- | --- | --- |
| Male gender | 0.64 [0.22-1.83] | *0.40* | *-* |  |
| Age (years) | 1.00 [0.97-1.03] | *0.89* | *-* |  |
| LVEF (%) | 0.98 [0.94-1.01] | *0.29* | *-* |  |
| Idiopathic dilated cardiomyopathy | 1.22 [0.42-3.48] | *0.70* | *-* |  |
| Ischemic cardiomyopathy | 2.85 [1.08-7.49] | *0.03* | 1.52 [0.54-4.23] | *0.42* |
| Hypertrophic cardiomyopathy | 0.37 [0.049-2.80] | *0.33* | *-* |  |
| ARVD | 0.82 [0.38- 2.61] | *0.87* | *-* |  |
| Ionic channel disorders | 0.34 [0.08- 1.67] | *0.56* | *-* |  |
| Hypertension | 1.98 [0.72-5.40] | *0.17* | - |  |
| Diabetes | 1.87 [0.69-5.08] | *0.21* | *-* |  |
| COPD | 0.35 [0.04-2.68] | *0.31* | *-* |  |
| CAD | 2.36 [0.91-6.13] | *0.07* | *-* |  |
| CKD | 4.72 [1.79-12.45] | *0.001* | 2.81 [0.98-8.05] | *0.05* |
| AF History | 1.80 [0.63-5.13] | *0.26* | - |  |
| Previous valve replacement | 10.52 [3.61-30.64] | *<0.0001* | 7.22 [2.34-22.22] | *0.0006* |
| Previous CABG | 0.73 [0.09-5.57] | *0.76* | - |  |
| S-ICD | 0.05 [0.007-0.44] | *0.006* | 0.07 [0.009-0.55] | *0.01* |

**Supplemental Table 4:** Association between ICD appropriate therapy and clinical covariates

|  | **Univariate Analysis**  **OR [95% CI]** | ***P*** | **Multivariate Analysis**  **OR [95% CI]** | ***P*** |
| --- | --- | --- | --- | --- |
| Male gender | 2-26 [0.89- 5.71] | *0.08* | *-* | *-* |
| Age (years) | 1.01 [0.99-1.03] | *0.16* | *-* | *-* |
| LVEF (%) | 0.97 [0.95-0.99] | *0.01* | *0.97 [0.94- 1]* | *0.11* |
| Idiopathic dilated cardiomyopathy | 1.46 [0.83- 2.55] | *0.19* | *-* | *-* |
| Ischemic cardiomyopathy | 1.56 [0.90- 2.71] | *0.11* | *-* | *-* |
| Hypertrophic cardiomyopathy | 0.59 [0.26- 1.32] | *0.19* | *-* | *-* |
| ARVD | 3.42 [1.22- 9,56] | *0.02* | *3.68 [0.79-17.14]* | *0.1* |
| Ionic channel disorders | 0.23 [0.05- 0.95] | *0.04* | *0.28 [0.1- 1.61]* | *0.22* |
| Atrial fibrillation | 1.33 [0.72-2.46] | *0.36* | *-* | *-* |
| Hypertension | 1.35 [0.77-2.37] | *0.29* | - | *-* |
| Diabetes | 1.77 [1.02- 3.09] | *0.04* | *1.58 [0.81-3.08]* | *0.18* |
| COPD | 0.21 [0.05- 0.85] | *0.03* | *0.33 [0.08-1.40]* | *0.13* |
| CAD | 1.23 [0.70- 2.15] | *0.48* | *-* | *-* |
| CKD | 1.53 [0.74- 3.14] | *0.25* | *-* | *-* |
| Previous Valve replacement | 1.65 [0.39- 6.90] | *0.49* | *-* | *-* |
| Previous CABG | 0.72 [0.22- 2.31] | *0.58* | *-* | *-* |
| S-ICD | 0.46 [0.21-0.98] | *0.04* | 0.54 [0.25- 1.18] | *0.12* |

|  | **Univariate Analysis**  **OR [95% CI]** | ***P*** | **Multivariate Analysis**  **OR [95% CI]** | ***P*** |
| --- | --- | --- | --- | --- |
| Male gender | 1.70 [0.57- 5] | *0.34* | *-* | *-* |
| Age (years) | 1.03 [0.99-1.06] | *0.1* | *-* | *-* |
| LVEF (%) | 1 [0.96-1.06] | *0.70* | *-* | *-* |
| Idiopathic dilated cardiomyopathy | 0.94 [0.39- 2.26] | *0.89* | *-* | *-* |
| Ischemic cardiomyopathy | 1.28 [0.57- 2.87] | *0.54* | *-* | *-* |
| Hypertrophic cardiomyopathy | 0.73 [0.1-5-51] | *0.76* | *-* | *-* |
| ARVD | * | *** | *-* | *-* |
| Ionic channel disorders | 0.51 [0.07- 3.87] | *0.52* | *-* | *-* |
| Atrial fibrillation | 0.92 [0.34-2.12] | *0.84* | *-* | *-* |
| Hypertension | 1.26 [0.57-2.77] | *0.57* | - | *-* |
| Diabetes | 1.23 [0.57- 2.67] | *0.60* | *-* | *-* |
| COPD | 2.51 [0.90- 6.98] | *0.08* | *-* | *-* |
| CAD | 1.30 [0.57- 2.97] | *0.53* | *-* | *-* |
| CKD | 0.55 [0.21- 1.42] | *0.21* | *-* | *-* |
| Previous Valve replacement | 4.17 [0.89- 19.42] | *0.07* | *-* | *-* |
| Previous CABG | 0.92 [0.35- 2.41] | *0.87* | *-* | *-* |
| S-ICD | 0.89 [0.38-2.09] | *0.79* | *-* | *-* |

**Supplemental Table 5:** Association between mortality and clinical covariates
